# Assessment of the Fruit Chemical Characteristics and Antioxidant Activity of Different Mulberry Cultivars (*Morus* spp.) in Semi-Arid, Sandy Regions of China

**DOI:** 10.3390/foods12183495

**Published:** 2023-09-20

**Authors:** Zhiyu Sun, Yongbin Zhou, Wenxu Zhu, You Yin

**Affiliations:** 1 Life Science and Technology College, Dalian University, Dalian 116622, China; szy101@stu.syau.edu.cn; 2 College of Forestry, Shenyang Agricultural University, Shenyang 110866, China; zhuwx@syau.edu.cn; 3 Institute of Modern Agricultural Research, Dalian University, Dalian 116622, China; 4 Research Station of Liaohe-River Plain Forest Ecosystem, Chinese Forest Ecosystem Research Network (CFERN), Shenyang Agricultural University, Tieling 110161, China

**Keywords:** mulberries fruit characteristics, total phenolics, antioxidant activity

## Abstract

As a traditional cash crop with ecological and nutritional values, mulberry is gradually expanding its consumption worldwide due to its great regional adaptability and superior health functions. The widespread interest in nutrients has led to a growing need to explore in depth the health benefits of mulberries. Many studies are actively being conducted to investigate the adaptability of the diversity of mulberries in different applications. This study systematically investigated the physicochemical properties and antioxidant activity of four mulberry genotypes cultivated in China’s semi-arid sandy regions to better understand the composition and health-promoting potential of this super crop. Chemical composition identification was identified via HPLC and antioxidant activity was further determined via DPPH and FRAP. The moisture, crude protein, ash, soluble solids, phenolics, anthocyanins, and flavonoids contents of mulberry were comparatively analyzed. The study revealed that the four mulberry genotypes showed significant differences in quality and content of the analyzed characteristics. The greatest antioxidant activity was found in Shensang 1, which had the most soluble solids (17%) and the highest amounts of free sugar (fructose: 5.14% and glucose: 5.46%). Ji’an had the most minerals (K: 2.35 mg/g, Ca: 2.27 mg/g, and Fe: 467.32 mg/kg) and it also contained chlorogenic acid, which has the potential to be turned into a natural hypoglycemic agent. PCA and Pearson correlation analysis indicated that the antioxidant activity was closely related to the chemical contents of total phenols, flavonoids, anthocyanins, and soluble sugars. If the antioxidant activity and nutrient content of the developed plants are considered, Shen Sang 1 is the most favorable variety. This finding can be used to support the widespread cultivation of mulberries to prevent desertification as well as to promote the development of the mulberry industry.

## 1. Introduction

As a perennial deciduous woody tree, the mulberry family (Moraceae) is widely distributed in all continents and is adaptable to various geological and climatic environments [1,2,3]. There are approximately 30–33 species of mulberry trees in the world, and China has 25 species, making it the worldwide distribution center of mulberry trees [4]. As a traditional cash crop, mulberry trees meet the growing economic and ecological needs of a society [5]. As early as ancient China, the “Compendium of Materia Medica” described many of the medicinal properties of the mulberry tree [6]. Today, as a medicinal food plant recognized by the Chinese Ministry of Health, the mulberry is an important biological resource and has formed a complete industrial chain in the fields of medicine, healthcare, feed, and food [7], providing employment for millions of people. Since the fruits, leaves, branches, and bark of the mulberry tree are used in the preparation of various products for the pharmaceutical, food, cosmetic, and healthcare industries, it is now being industrially developed [8,9]. In particular, the fruits of the mulberry tree are rich in nutrients and bioactive compounds with a variety of pharmacological properties, making it a potential disease-fighting food for the prevention or treatment of chronic diseases [10]. It is rich in proteins, lipids, carbohydrates, fiber, minerals (K, Ca, Fe, and Zn), and vitamins and is low in calories, making it a healthy food choice for consumers [11]. Health-conscious consumers are increasingly interested in choosing the foods with bioactive ingredients, as a health booster [12]. The sugar content of mulberry was reported to increase during ripening [13]. While the accumulation process of chemicals in plants is influenced by various factors such as genetics [14]. Khan et al. concluded that the antioxidant activity of *Morus alba* polysaccharides was significantly correlated with origin [15]. Liu et al. concluded that the DPPH radical and superoxide radical scavenging activities of polysaccharides in *C. paliurus* leaves significantly varied among different planted areas [16]. Mulberry is an important source of mineral elements, in particular, potassium and, to a lesser extent, calcium [17,18]. Sun et al. concluded that mineral element content interacted with sugar, crude protein, crude fat, and pectin content [19]. In particular, its black berries contain very important chemical substances with high anthocyanin contents and antioxidant activities, which are useful for the increases in primary agricultural production, the food industry, and the prevention and treatment of human diseases [20,21]. Aramwit et al. concluded that purple mulberry extracts had higher total sugar and anthocyanin contents than red and fuchsia mulberry extracts because of the need for sugar as a precursor for the synthesis of anthocyanins [22]. Mulberries are also rich in a variety of other chemical components such as 1-deoxynojirimycin, stilbenoids (such as resveratrol and piceatannol), flavonoids (such as quercetin, rutin, isoquercitrin, and cyaniding 3-glucoside), and phenolic acids (such as gallic, protocatechuic, and chlorogenic acid). Among these, rutin and quercetin not only possess a variety of biological activities but also have neuroprotective effects [23]. These phytochemicals have multiple hydroxyl groups on their aromatic rings, providing them with the antioxidant potential for scavenging free radicals, which is associated with disease prevention. It can be seen that mulberry represents a potential source of defense against oxidative stress-related diseases. Of course, antioxidant activities depend not only on phenolic contents but also on the properties of the phenolic compounds, the presence of other phytochemicals, and the synergistic effects between them [24,25]. Chen et al. reported that the phenolic compound contents in mulberries were higher than those in blackberries, blueberries, raspberries, and strawberries, indicating that mulberries can be a good source of phenolic compounds [26]. Yang et al. reported that the total phenolic, total flavonoid, and anthocyanin contents of freeze-dried mulberry powder were 23.0 mg/g gallic acid equivalent, 3.9 mg/g rutin equivalent, and 0.87 mg/g cyanidin-3-glucoside equivalent, respectively [8]. 

As a native tree species in China and combined with consideration of the current development prospects of the mulberry industry chain, we can conclude that it is the most suitable and beneficial plant for sustainable development. However, the diversity in the agro-climatic conditions, soil conditions, and geographical constraints affects the contents of various chemical components (including sugars, minerals, phenolic compounds, and antioxidant activity) in mulberries. Numerous studies in arid and semi-arid regions have shown that afforestation is one of the most popular and effective strategies for sand fixation to stop desertification [27,28]. On the southern edge of the Horqin Sand Region in China, northwestern Liaoning Province is surrounded by arid, persistently windy, water-scarce, and sparsely vegetated areas [29]. How can the incomes of local farmers be stabilized while restoring vegetation? The development of mulberry resources in extreme climate areas directly affects the quality of the fruit. To our knowledge, there have been many studies on mulberry plants, but there are few scientific data on the commercial aspects of the growth, nutritional status, and phytochemical qualities of various mulberry genotypes in semi-arid, sandy areas. This has prevented us from further understanding the suitability of each genotype in developing various applications. The aim of this study was to analyze four mulberry genotypes: A’erxiang (ARX), Fujia (FJ), Ji’an (JA), and Shensang 1 (SS). Two flavonoids (rutin and quercetin), two anthocyanin monomers (cyanidin-3-O-glucoside and peonidin-3-O-glucoside), and chlorogenic acid were detected using the HPLC-DAD technique. The antioxidant activities of the extracts were evaluated using DPPH and FRAP. The results of the study will provide a scientific basis for further investigation of the growth and quality characteristics of mulberries in windswept areas and for scientific screening and the development of northern mulberry germplasm resources.

## 2. Materials and Methods

### 2.1. Experimental Site

The study was conducted at the Mulberry Garden Experimental Farm in Changwu County, Fuxin City (122°49′ E, 42°35′ N), which is located at the southern edge of the Horqin Land. The altitude of the test site is about 87.7 m above sea level, the average annual temperature is 7.2 °C, and the average annual sunshine hours are 2822.6 h. It is windy, drought-prone, with rain and heat in the same season, and the regional ecological environment is fragile. The region belongs to the northern temperate zone semi-arid continental monsoon climate, the climate is characterized by drought and wind, located in the semi-arid agricultural and pastoral areas, annual precipitation in the range of 350–500 mm, extreme years have been in the 200 mm range or so, while the same period of evaporation is precipitation is 3 to 5 times. The wind period and drought period are synchronized, forming “nine droughts in ten years” [5,30].

### 2.2. Mulberry Sample Preparation

Mulberry cultivars suited for growing in northern China are limited. The standard mulberry cultivar Shensang 1 (variety approved by Shenyang Agricultural University) and three other mulberry genotypes (A’erxiang, Fujia, and Ji’an) were chosen for this study based on the climatic conditions of the semi-arid aeolian sandy terrain in northwest Liaoning. Morphological characteristics of Fujia: leaf blade small margin serrates, entire to deeply cleft, good cold resistance, no blight, strong fruiting ability, and medium fruit size. Morphological characteristics of Ji’an: leaf margin serrate, tooth apical awn is not obvious, purple–black fruit after maturity, and medium yield. Morphological characteristics of A’erxiang: fast growth, leaf margin serrate, entire, fruit purple–black, and high yield. Table 1 lists the key plant features of the cultivars and genotypes. The experimental seedlings were jointly planted in the mulberry planting resource breeding base in Zhangwu County, Fuxin City, and in 2021, at five years of age, they were used as research materials. The berries were manually harvested in the field to prevent any potential mechanical damage. They were instantly frozen in liquid nitrogen tanks and transported to the laboratory, where they were kept in a −20 °C freezer until needed. Each experimental analysis comprised four replicates.

### 2.3. Chemicals and Reagents

Rutin, catechin, gallic acid, resveratrol, KH_2_PO_4_, H_3_PO_4_, aluminum chloride, Folin–Ciocalteu reagent, chlorogenic acid, cyanidin-3-O-glucoside, peonidin-3-O-glucoside, and quercetin were all obtained from Aladdin Co., Ltd. (Shanghai, China). All reagents used in this research were analytically pure. The methanol, TFA, and acetonitrile used for the HPLC analysis were obtained from Dingguo Biological Reagent Co., Ltd. (Beijing, China). The antioxidant assay ELISA kits were obtained from Enzyme-linked Biotechnology Co., Ltd. (Shanghai, China).

### 2.4. Mulberry Conventional Quality Assay

The following fruit parameters were recorded using the method established by Mars et al. [31]: plant height (PH) (cm), diameter breast height (DBH) (cm), berry length (BL) (mm), berry width (BWI) (mm), berry weight (BWE) (g), and moisture content (MC) (%). AOAC [32] techniques were used to calculate the crude protein contents (CPC) (%) and ash contents. After dry digestion, the mineral elements (Ca, Fe, K, and Zn) were measured using a flame atomic spectrophotometer. The total soluble solids (TSS) (%) of the correctly weighed mulberry (5 g) were measured and reported as °Brix using a digital refractometer (Atago Co., Ltd., Tokyo, Japan).

### 2.5. Phytochemical Extraction of Mulberry

The mulberry extracts were prepared for the experiments on total anthocyanins, flavonoids, and phenolics. The mulberry extraction process was based on the procedure published by Lin et al. [33], with minor adjustments. In summary, 20 g of mulberries were precisely weighed and then crushed using liquid nitrogen in a mortar. The samples were extracted in an ultrasonic (SB25-12DTN, Ningbo Xinzhi Bio-technology Co., Ningbo, China) bath at 40 °C for 1 h with 100 mL of acidified ethanol (0.1% HCl). The extraction procedure was repeated until the solvent became transparent after the solution was vacuum-filtered. The supernatant was mixed and then evaporated at 40 °C using a rotary evaporator (RE-5203A, Shanghai Bilong Instruments Co., Ltd., Shanghai, China) until no alcohol remained. The recovered residues were fixed to 10 ml with acidified aqueous solution (pH = 3.0).

### 2.6. Total Anthocyanins Contents Assay (TAC)

The TAC differences between the mulberries were determined using the traditional pH differential technique [34]. We diluted 1 mL of sample with 9 mL of potassium chloride buffer (pH of 1.0) and 9 mL of sodium acetate buffer (pH of 4.5). After 20 min of incubation at room temperature, we measured the absorbance at 520 and 700 nm. The differences in absorbance were calculated using Equation (1) and the TAC values were computed using Equation (2) (to determine the quantity of cyanidin-3-glucoside/100 g FW) as follows:

∆A = (A520 − A700) × pH1.0 − (A520 − A700) × pH4.5,(1)

TAC = (∆A × MW × DF × 100)/(ε × l)(2)

where ΔA is the absorbance difference, DF is the dilution factor, MW is the molecular weight of the cyanidin-3-glucoside (449.20 g/mol), ε is the molar absorptivity of the cyanidin-3-O-glucoside (26,900 L mol^−1^·cm^−1^), and l is the path length (1 cm). The measurements were repeated in triplicate.

### 2.7. Total Phenolics Contents Assay (TPC)

The total phenolic contents of the concentrates were calculated using the method established by Singleton et al. [35]. After combining 1 mL of concentrate, 1 mL of Folin–Ciocalteu reagent (0.2 N), and 5 mL of deionized water, 3 mL of sodium carbonate solution (75 g/L) was added to the mixture. A UV-Vis (TU1810, Puxi, Beijing, China) spectrophotometer determined the absorbance at 765 nm after covering the example tube with aluminum foil for utes. Using gallic acid as a standard, the findings were expressed as mg of gallic acid counterparts (GAE)/100 g FW.

### 2.8. Total Flavonoid Contents Assay (TFC)

The total flavonoid contents were assessed using the aluminum nitrate technique [36], with rutin serving as the control material (with minor modifications). In brief, a 96-well plate was filled with 20 μL of mulberry extract solution and rutin standard solution and incubated for 5 min at room temperature after adding 80 μL of 30% ethanol-containing aqueous solution and 10 μL of 0.5 M NaNO_2_ solution. The mixture was incubated with 10 μL of 0.3 M AlCl_3_ solution and 40 μL of 0.1 M NaOH solution consecutively for at room temperature before the absorbance was measured at 510 nm. We plotted a standard curve with concentrations ranging from 50 to 500 μg/mL, and then we used the standard curve equation to calculate the total flavonoid contents in the samples.

### 2.9. HPLC-DAD

#### 2.9.1. Analysis of Flavonoid and Anthocyanin

The HPLC-DAD system (Agilent 1100, DAD G4212B) was used to analyze the separation of the anthocyanins and flavonoids. We weighed 5 mL of mulberry extract and added 2 mL of chromatographically pure methanol solution, and the mixture was filtered through 0.22 m filters for HPLC detection. A Dikma Spursil C18 column (4.6 mm, 250 mm, 5 m) was used as the chromatographic column. The temperature of the chromatographic column was 35 °C. Gradient elution was used to separate the various anthocyanins and flavonoids. A (0.1% TFA-water) and B (0.1% acetonitrile) made up the mobile phase, and 0.7 mL/min was the flow rate, with a 20 μL injection volume. The temperature of the autosampler was equal to room temperature. The gradient elution system was as follows: 0–45 min, 100–55% A, 0–45% B; 45–47 min, 55–100% A, 45–0% B; and 47–52 min, 100% A, 0% B. The anthocyanin and flavonoid contents were determined using the areas of the appropriate peaks and standard calibration curves. Real-time chromatograms of the anthocyanins and flavonoids were acquired using diode array detection at 520 nm and 350 nm, respectively. The anthocyanin content was determined by absorbance at 520 nm. The standard curves for P3O (y = 45.314x, R^2^ = 0.9976). The flavonoids content was determined by absorbance at 350 nm. The standard curves for rutin (y = 42.731x, R^2^ = 1), quercetin (y = 78.358x, R^2^ = 1), and C3O (y = 2.6283x, R^2^ = 1).

#### 2.9.2. Analysis of Phenolics

The HPLC-DAD system (Agilent 1100, DAD G4212B) was used to analyze the phenolic separation. We weighed 5 mL of mulberry extract and added 2 mL of chromatographically pure methanol solution, and the mixture was filtered through 0.22 m filters for HPLC detection. A Dikma Spursil C18 column (4.6 mm, 250 mm, 5 m) was used as the chromatographic column. The temperature of the chromatographic column was 30 °C. The various mulberry phenolics were then isolated using gradient elution. A (KH_2_PO_4_ 20 mmol/L, H_3_PO_4_ adjusted to a pH of 2.6) and B (methanol) comprised the mobile phase, and 0.8 mL/min was the flow rate. The sample injection volume was 10 μL. Furthermore, the temperature of the autosampler was the same as room temperature. The gradient elution system was as follows: 0–5 min, 98–95% A, 2–5% B; 5–40 min, 95–75% A, 5–25% B; 40–0 min, 75–70% A, 25–80% B; 50–55 min, 70–98% A, 80–2% B; and 55–60 min, 98% A, 2% B. The phenolic contents were determined using the matching peak areas and standard calibration curves. Real-time chromatograms of the catechin, gallic acid, and chlorogenic acid contents were collected at 278 nm and 320 nm by diode array detection. The phenolics contents were calculated by the corresponding peak areas and standard calibration curves. The standard curves for chlorogenic acid (y = 19.458x + 144.68, R^2^ = 0.9999).

### 2.10. Antioxidant Activity Assay

The ELISA (enzyme-linked immunosorbent assay) double antibody one-step sandwich technique was used to perform the tests. In a nutshell, the strips were removed from the foil bags after utes at room temperature. The standard and sample wells were filled with 50 μL of DPPH standard, 10 μL of the sample to be tested, and 40 μL of the sample diluent. In addition to the blank wells, we added 100 μL of horseradish peroxidase (HRP)-labelled detection antibody to each well of the standard and sample wells, sealed the reaction wells with a sealing film, and incubated them for at 37 °C in a water bath or incubator. We then discarded the liquid, patted them dry on absorbent paper, filled each hole with washing up liquid, let them stand, shook off the washing liquid, patted them dry on absorbent paper, and repeated the plate washing 5 times (the board may also have been washed in the plate washing machine). Substrates A (carbamide peroxide) and B (TMB color developer) (50 μL) were added to each well and shielded from light at 37 °C. We then added 50 μL of stop solution to each well and measured the OD value of each well at 450 nm afterwards. We drew the standard curves by using the standard concentrations as the abscissas and the associated OD values as the ordinates; then, we drew the standard products’ linear regression curves and calculated the concentration values of each sample using the curve equation. The DPPH solution had a solid linear connection, with absorbance measurements in the range of 0–480 pg/mL, the regression equation y = 278x − 8.2576, and the correlation value R^2^ = 0.9923. The method used to calculate the FRAP was compatible with DPPH. The solution had a satisfactory linear relationship with absorbance ranging from 0 to 24 pg/mL, the regression equation y = 15.86x − 0.9413, and the correlation value R^2^ = 0.9968.

### 2.11. HPLC Condition Used for Sugar Analysis

HPLC was used to identify the individual inverted sugars (glucose, fructose, and sucrose). The standard curve preparation was as follows: we separately weighed 2.0 g each of sucrose, glucose, and fructose, and weakened them in 100 mL volumetric flasks to create a single standard solution of 20 mg/mL, which was filtered with a 0.45 μm filter membrane for the HPLC analysis. The single standard solution was blended to create a mixed standard solution, which was then diluted into six mixed standard solutions with different concentration gradients in the following order: 20 mg/mL, 10 mg/mL, 5 mg/mL, 2.5 mg/mL, 1.25 mg/mL, and 0.625 mg/mL. The injection volume injected was 15 μL. We used the linear regression equation to calculate the peak areas and concentrations with the abscissas and ordinates of the standard curves. The sample preparation was as follows: we took 2.0 g of mulberries, added 10 mL of distilled water to the extracts, centrifuged them to collect the supernatants, filtered them through 0.45 μm filters, and then analyzed them to create sample solutions. The injection volume was 15 μL. The standard curves were used to convert the contents of each soluble sugar. The chromatographic conditions included an XBridge Amide column (250 mm × 4.6 mm × 5 μm), an HPLC with an RID detector, a mobile phase comprising acetonitrile–water (75:25, *v*/*v*) with a flow rate of 1.0 mL/min, and a column temperature of 35 °C.

### 2.12. Statistical Analysis

The analyses included four replicates. The data were reported as means and standard deviations and analyzed using a one-way analysis of variance (ANOVA) test, followed by a Duncan test. The SPSS 25 program was utilized for the statistical analysis based on the parameter test with a significance level of 0.05 (two-tailed *p* value). Pearson correlation analysis was used to investigate the link between the chemical composition and the antioxidant activity of the mulberry extracts, and principal component analysis (PCA) was utilized to assess the differences across the mulberry samples using Origin 2021. 

## 3. Results and Discussion

### 3.1. Nutritional Indicators of Four Mulberry Genotypes

#### 3.1.1. Mulberry Conventional Quality

**Figure 1:**
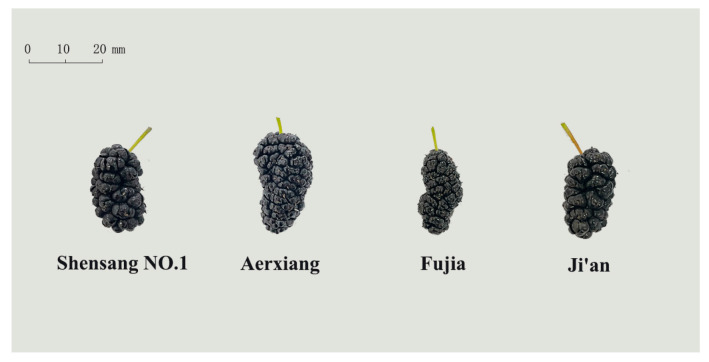
Images of four mulberry genotypes.

The fresh mulberries are shown in Figure 1. The BL range of the four mulberries was 22.23–27.71 mm, the BWI range was 11.73–12.93 mm, and the BWE range was 2.62–3.14 g, with A’erxiang berries having the greatest sizes and weights. A fruit’s moisture percentage is a key factor for determining its shelf life and edible acceptability. The freshness and the moisture level of a fruit determine how quickly it will degrade. The results were similar to those obtained by Nayab et al. [37], who found MCs in the range of 76.07–89.87% across four mulberry genotypes in Pakistan. In the present study, though Fujia was the smallest berry it was also the moistest. Berry size is a changeable property that varies based on environmental and agricultural factors [38]. Berry measurement and data collection are critical for the preservation of these mulberry genotypes [39].

Table 2 shows the findings for the CPC, TSS, ash, mineral elements, and free sugar determinations of the assorted mulberries. The CPCs of the four mulberries varied from 1.24% to 1.77%, with Fujia and A’erxiang having the highest CPCs. The ash percentages of the four mulberries varied from 0.77% to 1.12%, with Ji’an having the highest ash concentration, followed by A’erxiang, Shensang 1, and Fujia. The ash content values were greater than those found by Nayab et al. [37], who found ash concentrations in mulberry fruits ranging from 0.35% to 0.81%. The TSS contents differed between the samples in a somewhat significant way. The TSS concentrations of the four mulberries varied from 8.99% to 17%, with Shensang 1 having the highest TSS percentage. 

In terms of mineral elements, the Zn contents of the four mulberries ranged from 27.22 mg/kg to 28.63 mg/kg, although there was no significant difference, and Fujia had the lowest K, Ca, and Fe contents while Ji’an has the greatest K and Ca contents. The K contents of the four mulberries ranged from 1.94 mg/g to 2.35 mg/g, with Ji’an having the highest K content and Fujia the lowest. The Ca contents ranged from 1.39 mg/g to 2.27 mg/g, with Ji’an having the highest Ca content and Fujia the lowest. The Fe contents ranged from 347.2 mg/kg to 470.78 mg/kg, with Shensang 1 having the highest Ca content and Fujia the lowest. The K and Ca contents were the most abundant, with Fe and Zn following close after. The macro- and micro-element concentrations were in the following order: K > Ca > Fe > Zn. This was consistent with the findings of Paunovi et al. [21]. Sun et al. concluded that Ca was one of the most critical mineral elements, followed by K, Fe, and Zn [18]. Thus, mulberries are valuable edible berries with rich nutritional composition. The M. alba fruit has been demonstrated in past studies to be a great source of protein, as well as certain vital minerals, including calcium and potassium [40]. It has more protein than raspberries and strawberries [41,42]. Sugar content is an important indicator of fruit quality and taste development [43]. Sucrose was not present in our experimental samples, which was consistent with the findings of previous studies [43]. The amounts of glucose and fructose were comparable, with glucose being slightly superior, which was consistent with the findings of Eyduran et al. and Gundogdu et al. [44,45]. Both sugars had the highest Shensang 1 contents and the lowest Fujia contents. The fructose concentrations ranged from 2.04 (0.05%) to 5.14 (0.09%), whereas the glucose contents ranged from 2.04% to 5.46%. Among them, Shenzang 1 had the highest content, and Fujia had the lowest.

#### 3.1.2. Phenolics and Their Metabolites Mulberries

It is critical to choose mulberry cultivars that have high phenolic contents. HPLC was used to investigate the cyanidin-3-O-glucoside, peonidin-3-O-glucoside, rutin, chlorogenic acid, and quercetin contents, with all parameters documented in Table 3. The HPLC-DAD chromatogram of mulberry was shown in the Figure S1. Due to variables such as genetype and origin variances, the concentrations of resveratrol, gallic acid, and catechin in the samples were exceedingly low or even undetectable throughout the sample testing process. The secondary metabolite concentrations of the four mulberries differed substantially (Table 3). Rutin, C-3-O-glucoside, and P-3-O-glucoside were the most abundant in Shensang 1. Rutin’s Shensang 1 concentrations were 34.73% greater than those in Ji’an, which had the lowest concentrations. A’erxiang had higher quercetin concentrations than the other three mulberry genetypes, and they were 2.85 times higher than those of Ji’an, which had the lowest. The chlorogenic acid concentrations of Ji’an were greater than those of the other three mulberry genetypes and 0.97 times higher than those of Shensang 1. The results of the study yielded significant differences in the metabolite content of the examined mulberries, which is similar to the results of the study by Gundogdua et al. [17]. The differences may originate from genetic and environmental factors as well as cultural practices. Plant phenolics have been proven in several studies to have various bioactive properties, such as antioxidant activity, anti-diabetic potential, etc. [46,47]. Mature mulberries contain high concentrations of anthocyanins, primarily C-3-O-glucoside [48]. The structure of anthocyanin molecules is simple and easily absorbed, with antioxidant, anti-inflammatory, and chemoprotective effects that reduce the risk of cardiovascular disease and cancer, with the ability to scavenge free radicals and prevent free radical damage [49]. Rutin is one of the main active flavonoids that can reduce capillary fragility and improve microcirculation and can treat diabetes, hypertension, hyperglycemia, and other diseases [50]. Quercetin and catechins and other polyphenols can improve host obesity associated with high-fat diets by modulating the gut microbiota [51,52]. Chlorogenic acid has been reported to exert anti-obesity effects through beneficial regulation of intestinal flora, especially improving lipid and glucose metabolism [53,54].

### 3.2. Antioxidant Activity of Four Mulberry Genetypes

Antioxidant activity, as an important indicator for evaluating the health benefits of food products, can be determined using two methods that complement each other to a certain extent and are fast, simple, low-cost, and reproducible ways to measure the antioxidant potential of plant extracts [55]. The four mulberries’ DPPH scavenging activities varied from 239.69 pg/g to 294.86 pg/g (Table 4), with the maximum activity being 23.02% higher than the lowest. The capacity of DPPH to scavenge free radicals differed significantly between the genetypes. The DPPH free radical scavenging capacities of the Shensang 1 and A’erxiang samples were the highest, followed by Fujia and Ji’an. FRAP is another popular method for evaluating antioxidant activity. The four mulberries’ FRAPs varied from 159.12 pg/g to 249.86 pg/g, with the greatest antioxidant activity being 57.03% higher than the lowest. The FRAP free radical scavenging capacities of the various mulberries varied dramatically. The Shensang 1 sample has the highest FRAP free radical scavenging capacity, followed by A’erxiang, Ji’an, and Fujia. Antioxidants are compounds that stabilize, scavenge, and inhibit the production of oxidants and free radicals. Therefore, consumption of natural resource-based antioxidants (vegetables, fruits, and herbs) may help protect against oxidants and free radicals without side effects [56].

### 3.3. TAC, TPC, and TFC of Four Mulberry Genetypes

In-depth investigation of the chemical composition of mulberry can help develop and identify new natural antioxidant resources. Flavonoids, anthocyanins, and phenolics are abundant in many plants, and these active elements are crucial in a variety of pharmacological actions. In the present study, as indicated in Table 4, Shensang 1 had the highest TAC (567.6 mg/100 g FW), TFC (1.10 mg/100 g FW), and TPC (11.87 GAE/100 g FW). Conversely, Fujia had the lowest TAC (300.16 mg/100 g FW), A’erxiang had the lowest TFC (0.66 mg/100 g FW) and Ji’an had the lowest TPC (9.92 GAE/100 g FW). Farahani et al. discovered that the total anthocyanin contents of mulberries ranged from 25.67 to 569.69 mg/100 g FW, which was compatible with the findings of this study [57]. As demonstrated in Table 4, the Shensang 1 had 0.89 times the TAC of the lowest Fujia, 0.67 times the TFC of the lowest A’erxiang, and 0.20 times the TPC of the lowest Ji’an. In conclusion, the phenolic contents of the mulberries vary according to species, genetype, plant age, cultural and growing circumstance, extraction procedure, etc. [58]. Mulberries have more anthocyanins than blackberries, blueberries, blackcurrants, and redcurrants according to the study by Veberic et al. [59]. Mulberry phenolics may have a considerable effect on free radical scavenging [60]. Determining the phenolic contents (TPC), flavonoid contents (TFC), and anthocyanins contents (TAC) of plants is important.

### 3.4. Principal Component Analysis (PCA) and Pearson’s Correlation

**Figure 2:**
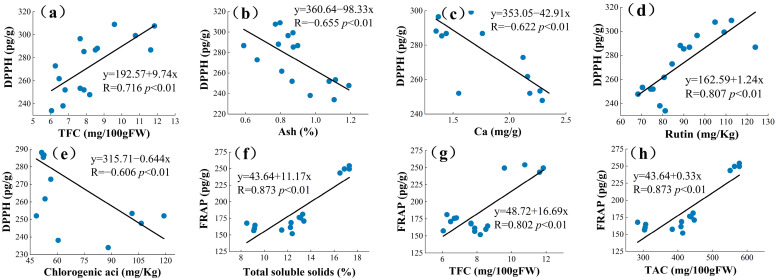

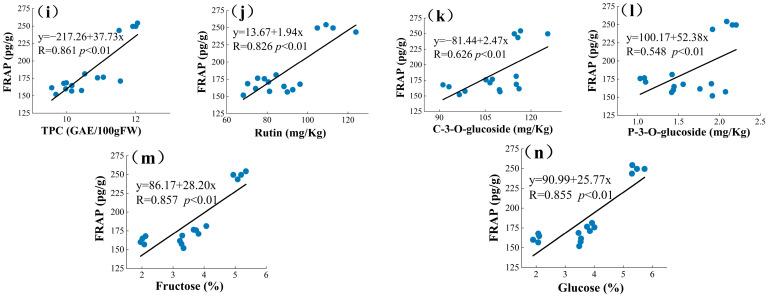
The correlation coefficient among phytochemical profile and antioxidant activities. Among them, (**a**–**e**) is the correlation between DPPH and each indicator, and (**f**–**n**) is the correlation between FRAP and each parameter.

The current ways to improve the quality of mulberry and improve the nutrition of berries are relatively limited, and an in-depth study of the interrelationship between the chemical and mineral components of mulberry can help explore new effective measures [17]. PCA is a statistical tool used to discover correlations between variables in order to validate links between characteristics and components [61]. PCA analysis may be used to correlate biochemical molecules [62]. A new variable is known as the main component (PC) and is used to decrease the quantity of raw data that must be evaluated [63]. Principal component analysis was used in this study to determine the relationships between the four mulberry genetypes (TSS, TAC, TFC, TPC, rutin (RU), quercetin (Qur), C-3-O-glucoside (C3O), P-3-O-glucoside (P3O), chlorogenic acid (CA), fructose (Fru), glucose (Glu), DPPH, and FRAP). These variables were summed up into a collection of uncorrelated variables, each of which was a linear combination of the major variables. In PCA, to correctly assess the main components, the variables must be connected with each other [64]. As a result, the linear relationship was calculated between different groups of phytochemical profiles and antioxidant activities of mulberry extracts using Pearson’s correlation prior to the principal component analysis (Figure 2). Higher correlation coefficient values suggested a greater contribution by the phytochemical profile to the antioxidant activities of the mulberry extracts. DPPH values showed strong correlation with TFCs (R = 0.716, *p* < 0.01), Figure 2a; Ash (R = −0.655, *p* < 0.01), Figure 2b; Ca (R = −0.622, *p* < 0.01), Figure 2c; Rutin (R = 0.807, *p* < 0.01), Figure 2d; and Chlorogenic acid (R = −0.606, *p* < 0.01), Figure 2e. Significantly high correlations were also found between FRAP with TSS (R = 0.873, *p* < 0.01), Figure 2f; TFCs (R = 0.802, *p* < 0.01), Figure 2g; TAC (R = 0.873, *p* < 0.01), Figure 2h; TPC (R = 0.861, *p* < 0.01), Figure 2i; Rutin (R = 0.826, *p* < 0.01), Figure 2j; C3O (R = 0.626, *p* < 0.01), Figure 2k; P3O (R = 0.548, *p* < 0.01), Figure 2l; Fructose (R = 0.857, *p* < 0.01), Figure 2m; and Glucose (R = 0.855, *p* < 0.01), Figure 2n. Plants have the ability to produce hundreds of organic molecules. Starting with fundamental pathways, certain secondary metabolite productions may arise from the same precursors and are, therefore, intimately related [65,66]. The above findings indicate that the antioxidant activity of the mulberries might be attributed to these active ingredients. Yao et al. concluded a significant correlation between the total phenolic content of mulberry seeds and DPPH and FRAP (r = 0.965 and r = 0.951) [63].

**Figure 3:**
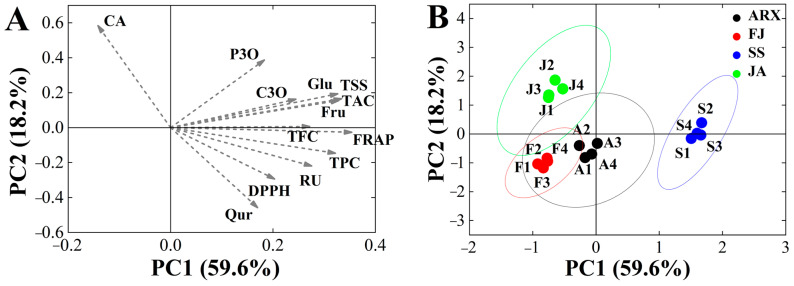
(**A**) PCA (PC 1 and PC 2) of mulberry genetypes including loading plots for TPC, TAC, TFC, antioxidant activity, and free sugar. (**B**) PCA score plot for four genetypes. Abbreviations: CA, Chlorogenic acid; C3O, C-3-O-glucoside; P3O, P-3-O-glucoside; RU, Rutin; Qur, Quercetin; TFC, the flavonoid content; TPC, the phenolics content; TAC, the anthocyanins content; Glu, Glucose; Fru, Fructose; TSS, Total soluble solids; SS, Shensang 1; ARX, Aerxiang; FJ, Fujia; JA, Ji’an.

The Kaiser–Meyer–Olkin value was 0.706 and the level of significance was 0.000, indicating that these data could be analyzed via PCA. Three principal components were identified using the principal component analysis, accounting for 91.27% of the variation in the overall dataset. PC1 was associated with the traits FRAP, TSS, TAC, Fru, Glu and TPC; PC2 was associated with CA, DPPH, and Rutin; and PC3 was associated with P3O and CA (Table 5). Figure 3B shows the score chart of the PCA graph, with each point representing a sample from each mulberry genetype. The findings revealed considerable geographical variances. It was discovered that Shensang 1 had greater Rutin, TAC, TFC, and TPC levels, as well as increased antioxidant activity, making it more acceptable for marketing as a type of health product. A’erxiang and Fujia were substantially close, indicating that the active component levels of the mulberries were similar in these genetypes. Ji’an had the greatest positive score for PC2, indicating that Ji’an, with its high CA concentrations, might potentially be used as a natural hypoglycemia product.

## 4. Conclusions

The mulberry tree is a medicinal and food-related plant that has been named “one of the top ten health foods of the twenty-first century”. The results revealed that Shensang 1 had a higher content of active compounds than the other genetypes, was more environmentally adaptable, and could be recommended for widespread cultivation as a commercial variety. Ji’an had a significant chlorogenic acid concentration and may be a natural source of chlorogenic acid. These findings demonstrated that mulberries have superior nutritional characteristics in harsh climatic environments, providing a foundation for developing corresponding breeding strategies and promoting the cultivation of high-nutrient and antioxidant-active mulberry resources in arid areas with wind and sand. Further studies on mulberry will reveal other unknown biological activities and help scientists to find new functional foods in natural plants. Of course, a more thorough investigation of the biology and pharmacology of the region’s mulberry resources is urgently required, and the value of cultivating mulberries in desolate places is immeasurable.

## Figures and Tables

**Table 1:** Mulberries conventional quality.

Geno Types	Origin	Location	Yield (kg/hm^2^)	PH (cm)	DBH (cm)	BL (mm)	BWI (mm)	BWE (g)	MC (%)
**Shen** **sang ^1^**	Huludao, LN	120°51′ E, 40°45′ N	71.70	235.67 ± 6.28 ^a^	12.50 ± 0.7 ^a^	23.81 ± 1.16 ^bc^	12.25 ± 0.49 ^a^	3.11 ± 0.01 ^a^	78.83 ± 0.1 ^c^
**A’er** **xiang**	Fuxin, LN	122°25′ E, 42°49′ N	61.33	179.00 ± 7.3 ^c^	11.12 ± 1.08 ^ab^	27.71 ± 0.87 ^a^	12.72 ± 1.04 ^a^	3.14 ± 0.02 ^a^	84.59 ± 0.52 ^b^
**Fujia**	Tieling, LN	123°48′ E, 43°17′ N	67.98	177.44 ± 7.16 ^c^	8.83 ± 1.06 ^b^	22.23 ± 1.02 ^c^	12.93 ± 0.68 ^a^	2.62 ± 0.04 ^b^	87.02 ± 0.15 ^a^
**Ji’an**	Tonghua, JL	125°92′ E, 41°49′ N	65.29	196.50 ± 6.56 ^b^	13.79 ± 1.32 ^a^	24.81 ± 0.85 ^b^	11.73 ± 0.82 ^ab^	3.06 ± 0.03 ^a^	83.49 ± 0.08 ^b^

^1^ Results are expressed as mean ± SD (n = 4). Values of each column with no letters in common are significantly different (*p* < 0.05). Abbreviations: PH, plant height; DBH, diameter breast height; BL, berry length; BWI, berry width; BWE, berry weight; MC, moisture content.

**Table 2:** Nutritional parameters of four mulberry genotypes.

Genotypes	CPC (%)	Ash (%)	TSS (%)	Mineral Elements				Free Sugars	
				K (mg/g)	Ca (mg/g)	Fe (mg/kg)	Zn (mg/kg)	Fru (%)	Glu (%)
**Shen** **sang ^1^**	1.53 ± 0.07 ^b^	0.83 ± 0.03 ^b^	17 ± 0.19 ^a^	2.05 ± 0.09 ^b^	1.81 ± 0.07 ^b^	470.78 ± 13.67 ^a^	27.22 ± 0.62 ^a^	5.14 ± 0.09 ^a^	5.46 ± 0.1 ^a^
**A’er** **xiang**	1.7 ± 0.08 ^a^	0.83 ± 0.06 ^b^	13.21 ± 0.09 ^b^	1.97 ± 0.12 ^b^	1.98 ± 0.14 ^b^	378.01 ± 12.25 ^b^	27.61 ± 2.14 ^a^	3.83 ±0.05 ^b^	3.89 ± 0.05 ^b^
**Fujia**	1.77 ± 0 ^a^	0.77 ± 0.06 ^b^	8.99 ± 0.16 ^d^	1.94 ± 0.02 ^b^	1.39 ± 0.02 ^c^	347.2 ± 12.4 ^b^	28.63 ± 1.36 ^a^	2.04 ± 0.05 ^d^	2.04 ± 0.05 ^d^
**Ji’an**	1.24 ± 0.04 ^c^	1.12 ± 0.02 ^a^	12.11 ± 0.21 ^c^	2.35 ± 0.03 ^a^	2.27 ± 0.04 ^a^	467.32 ± 13.05 ^a^	27.57 ± 2.1 ^a^	3.28 ± 0.02 ^c^	3.51 ± 0.02 ^c^

^1^ Results are expressed as mean ± SD (n = 4). Values of each column with no letters in common are significantly different (*p* < 0.05). Abbreviations: CPC, crude protein content; TSS, total soluble solids; Fru, fructose; Glu, glucose.

**Table 3:** Contents of C-3-O-glucoside, P-3-O-glucoside, rutin, quercetin, and chlorogenic acid of four mulberry genetypes.

Genetypes	Rutin (mg/kg)	Quercetin (mg/kg)	Chlorogenic Acid (mg/kg)	C-3-O-Glucoside (mg/kg)	P-3-O-Glucoside (mg/kg)
**Shensang ^1^**	112.63 ± 3.09 ^a^	13.11 ± 1.06 ^b^	52.55 ± 0.42 ^b^	118.08 ± 2.5 ^a^	2.09 ± 0.06 ^a^
**A’erxiang**	79.75 ± 1.91 ^c^	15.55 ± 0.63 ^a^	54.67 ± 2.55 ^b^	108.58 ± 2.23 ^ab^	1.16 ± 0.09 ^a^
**Fujia**	91.82 ± 1.74 ^b^	9.73 ± 0.71 ^c^	52.99 ± 0.9 ^b^	100.96 ± 3.1 ^b^	1.46 ± 0.03 ^b^
**Ji’an**	73.51 ± 2.85 ^c^	4.04 ± 1.03 ^d^	103.78 ± 2.38 ^a^	106.56 ± 4.27 ^ab^	1.91 ± 0.06 ^a^

^1^ Results are expressed as mean ± SD (n = 4). Values of each column with no letters in common are significantly different (*p* < 0.05).

**Table 4:** TAC, TPC, TFC, and the antioxidant activity (DPPH and FRAP) of four mulberry genetypes.

Genetypes	DPPH (pg/g)	FRAP (pg/g)	TAC (mg/100gFW)	TFC (mg/100gFW)	TPC (GAE/100gFW)
**Shensang ^1^**	294.86 ± 3.89 ^a^	249.86 ± 1.88 ^a^	567.6 ± 3.43 ^a^	1.10 ± 0.05 ^a^	11.87 ± 0.12 ^a^
**A’erxiang**	289.19 ± 2.5 ^a^	177.02 ± 1.97 ^b^	441.02 ± 2.99 ^b^	0.66 ± 0.01 ^c^	11.01 ± 0.22 ^b^
**Fujia**	250.22 ± 4.44 ^b^	159.12 ± 2.14 ^c^	300.16 ± 2.47 ^d^	0.82 ± 0.02 ^b^	10.04 ± 0.06 ^c^
**Ji’an**	239.69 ± 3.42 ^b^	159.57 ± 2.07 ^c^	404.53 ± 3.9 ^c^	0.74 ± 0.05 ^b c^	9.92 ± 0.19 ^c^

^1^ Results are expressed as mean ± SD (n = 4). Values of each column with no letters in common are significantly different (*p* < 0.05). Abbreviations: TAC, Total anthocyanins contents; TFC, Total flavonoid contents; TPC, Total phenolics contents.

**Table 5:** Principal component characteristic loadings of phytochemical composition and antioxidant activities.

	PC1 62.86%	PC2 17.33%	PC3 11.08%
**DPPH ^1^**	0.559	−0.730	0.260
**FRAP**	0.984	−0.127	0.025
**Total soluble solids**	0.931	0.328	−0.128
**Total anthocyanins contents**	0.931	0.328	−0.128
**Total flavonoid contents**	0.771	−0.290	0.433
**Total phenolics contents**	0.875	−0.090	−0.406
**Rutin**	0.763	−0.547	0.148
**C-3-O-glucoside**	0.695	0.215	0.018
**P-3-O-glucoside**	0.559	0.200	0.735
**Chlorogenic acid**	−0.345	0.767	0.520
**Fructose**	0.913	0.339	−0.174
**Glucose**	0.914	0.366	−0.118

^1^ PC1 represents 62.86% of the variance, PC2 represents 17.33% of the variance, and PC3 represents 11.08% of the variance.

## Data Availability

The data presented in this study are available on request from the corresponding author. The data are not publicly available due to the policy of the institute.

## Supplementary Materials

The following supporting information can be downloaded at: https://www.mdpi.com/article/10.3390/foods12183495/s1, Figure S1: HPLC-DAD chromatogram of mulberries.
