# Supplementary material for: Assessment of the Fruit Chemical Characteristics and Antioxidant Activity of Different Mulberry Cultivars (Morus spp.) in Semi-Arid, Sandy Regions of China
Source: Foods. 2023 Sep 20;12(18):3495. doi: 10.3390/foods12183495 (PMC10529437; doi:10.3390/foods12183495)
Supplement: Supplementary file 1 [file foods-12-03495-s001.zip › foods-2575433-supplementary.pdf]

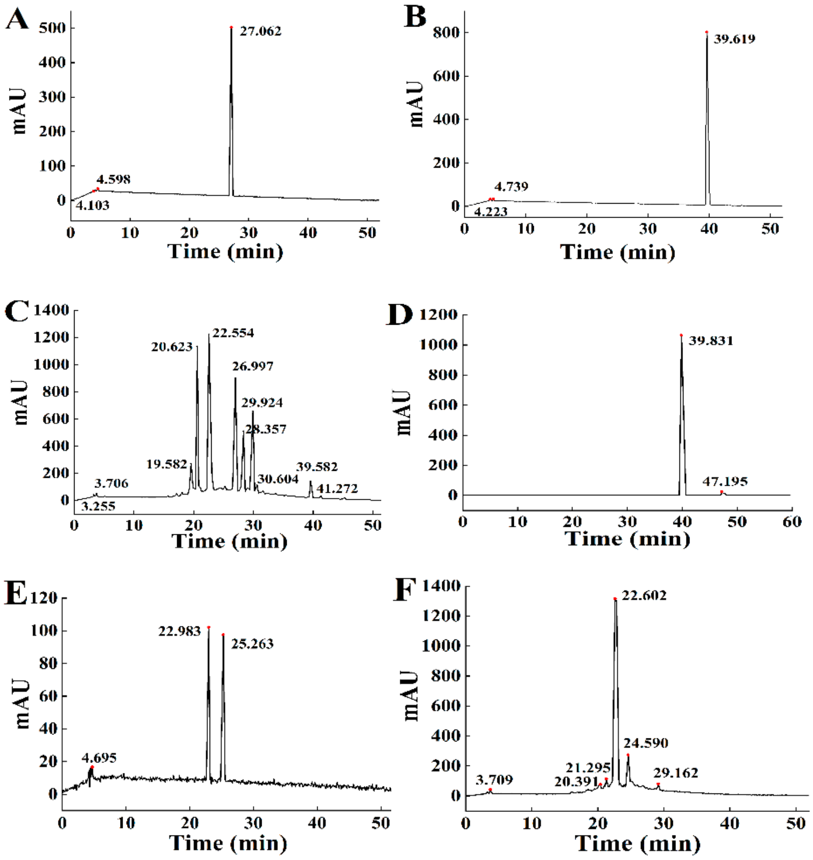

Figure S1. HPLC-DAD chromatogram of mulberries. Rutin (A), Quercetin (B), and Flavonoid (C) at 350 nm wavelength, Chlorogenic acid (D) at 320 nm wavelength, C-3-O-glucoside (E), P-3-O-glucoside (E), and Anthocyanins (F) at 520 nm wavelength
